# Causes of accidental childhood deaths in China in 2010: A systematic review and analysis

**DOI:** 10.7189/jogh.05.010412

**Published:** 2015-06

**Authors:** Kit Yee Chan, Xin–wei Yu, Jia–peng Lu, Alessandro Rhyll Demaio, Kirsty Bowman, Evropi Theodoratou

**Affiliations:** 1 The University of Edinburgh Medical School, Edinburgh, Scotland, UK; 2 Nossal Institute for Global Health, Melbourne School of Population and Global Health, University of Melbourne. Melbourne, Australia; 3 Municipal Key Laboratory of Clinical Epidemiology, School of Public Health, Capital Medical University, Beijing, China; 4 Harvard Global Equity Initiative, Harvard Medical School, Cambridge, MA, USA; 5 Copenhagen School of Global Health, University of Copenhagen, Copenhagen, Denmark

## Abstract

**Background:**

Infectious causes of childhood deaths in the world have decreased substantially in the 21st century. This trend has exposed accidental deaths as an increasingly important future challenge. Presently, little is known about the cause structure of accidental childhood deaths in low– and middle–income country (LMIC) settings. In this paper, we aim to establish cause structure for accidental deaths in children aged 0–4 years in China in the year 2010.

**Methods:**

In this paper, we explored the database of 208 multi–cause child mortality studies in Chinese that formed a basis for the first published estimate of the causes of child deaths in China (for the year 2008). Only five of those studies identified specific causes of accidental deaths. Because of this, we searched the Chinese medical literature databases CNKI and WanFang for single–cause mortality studies that were focused on accidental deaths. We identified 71 further studies that provided specific causes for accidental deaths. We used epidemiological modeling to estimate the number of accidental child deaths in China in 2010 and to assign those deaths to specific causes.

**Results:**

In 2010, we estimated 314 581 deaths in children 0–4 years in China, of which 31 633 (10.1%) were accidental. Accidental deaths contributed 7240 (4.0%) of all deaths in neonatal period, 8838 (10.5%) among all post–neonatal infant deaths, and 15 554 (31.7%) among children with 1–4 years of age. Among four tested models, the most predictive was used to establish the likely cause structure of accidental deaths in China. We estimated that asphyxia caused 9490 (95% confidence interval (CI) 8224–11 072), drowning 5694 (95% CI 5061–6327), traffic accidents 3796 (95% CI 3163–4745), poisoning 3163 (95% CI 2531–3796) and falls 2531 (95% CI 2214–3163) deaths. Based on medians from a few rare studies, we also predict 633 (95% CI 316–1265) deaths to be due to burns and 316 (95% CI 0–633) due to falling objects. Together, these 7 causes explain more than 80% of all accidental deaths when modeling is primarily used, and more than 95% when the analysis is based purely on medians from the 76 available studies.

**Conclusions:**

Reduction in global child mortality is a leading political priority and accidental deaths will soon emerge as one of the main challenges. In this paper we provided a detailed breakdown of causes of these deaths in a large middle–income country. We noted that, wherever the share of accidental deaths among all child deaths is increased, drowning is more likely to be the leading cause; asphyxia seems to be equally important in all contexts, while traffic accidents, poisoning and falls are relatively more important in contexts where the overall share of accidents to all child deaths is low.

Infectious causes of childhood deaths in the world have decreased substantially in the 21st century [1]. The World Health Organization (WHO), UNICEF and Child Health Epidemiology Reference Group (CHERG) estimated that the number of child deaths globally decreased from about 10.8 million in the year 2000 to about 7.6 million in the year 2010 and the majority of the reduction is attributable to fewer deaths from common childhood infections, such as pneumonia and diarrhoea [2,3]. This trend has exposed accidental deaths as an increasingly important future challenge. In 2000 accidents were estimated to contribute 3% to the total number of child deaths globally and in 2010 this increased to 5% [2,3]. In China, as perhaps the best example of a large middle–income country, these trends were even more pronounced. The child mortality decreased by nearly 75% between 2000 and 2010 and CHERG estimated that the proportion of accidental deaths in China increased from 9% to 11% during the same period [4]. To address the emerging challenge of childhood accidental deaths, more information is required on the specific causes and patterns of their occurrence in different contexts. Presently, little is known about the cause structure of accidental childhood deaths in low– and middle–income country (LMIC) settings.

In 2008, the WHO estimated up to 950 000 fatalities from injuries for children aged 0–18 years globally [5]. In the same year, WHO and UNICEF published their landmark *World report on child injury prevention* [6]. It was estimated that some 90% of these deaths were attributable to unintentional injuries [5] and that 95% of injury–related deaths in children occurred in LMIC [5,6]. The recent WHO's *World Health Statistics 2013* shows that the percentage distribution of injuries for the under five mortality has increased across all sub–categories by age and income from 2000 to 2010 [5–7]. However, information on fatal injuries from LMIC is often derived from medical facilities, thus underestimating the population–based burden [8]. This problem is made worse because definitions for injury mechanisms have not been uniform across different study settings. They can particularly vary with regards to drowning, burns, poisonings, and what is defined as “other unintentional injuries” [9–11].

There is persisting uncertainty over the estimates of accidental child deaths globally. In many low and middle-income regions, accidents are relatively rare among other causes of child deaths, contributing only several percents. This makes them prone to greater over– or under–estimation in multi–cause models, such as those used by CHERG, in comparison to the more frequent causes of death. Recently, an increased number of single–cause studies focused on accidents as causes of deaths in developing countries have been published, which is especially true for China. Previous results from a multi–cause model from China have already suggested a possible under–estimation of accidents and injuries as a cause of child deaths globally [4]. Better understanding of specific cause composition of accidental childhood deaths would reaffirm previous reports and assist in developing strategies and policies that could prevent these deaths in different LMIC contexts.

This study has four aims: (1) To estimate the total number of accidental deaths in children under five years in 2010 among neonates, post–neonatal infants, and children aged 1–4 years in all Chinese provinces; (2) to estimate the relative contribution of accidents to all child deaths in each of these age groups; (3) to define a specific cause to all accidental childhood deaths in China; and (4) to investigate if there are any significant context–related predictors of the proportional contribution of specific causes to the overall number of deaths due to accidents.

## METHODS

In terms of methods, this study is an extension of our previous study on the causes of child deaths in China in 2008, where a systematic review of Chinese literature was performed and then followed by epidemiological modeling [4]. In that study, we identified 206 multi–cause studies published between 2000 and 2008 that contributed information on a very large number of childhood deaths (about 350 000 in total) that had an exact cause assigned. That information allowed us to develop models that related the proportional contribution of each major cause of child deaths to the underlying context–specific under–five mortality rate (U5MR) [4]. If the U5MR for the whole of China is known, then the same models can be applied to estimate the proportional contribution of different causes to all child deaths in China in any given calendar year for which U5MR is available. Moreover, if the number of livebirths is also available for the same calendar year of interest, then the U5MR and proportions attributable to different causes can be translated into absolute number of deaths due to each cause [4].

To address *the first aim* of this study – ie, to estimate the total number of accidental deaths in children under five years of age in China in 2010 among neonates, post–neonatal infants, and children aged 1–4 years in all Chinese provinces – we needed to obtain the U5MR for China in the year 2010. This was available from the CHERG national–level estimate of the causes of child deaths for the year 2010 [3]. Using the same models as in our previous paper on the causes of child deaths in China in 2008, we estimated the proportional cause contribution in the year 2010 by province and 3 major age groups: neonates (0–28 days), post–neonatal infants (1–11 months), and children aged 1–4 years. We presented the results in **Table 1****,** showing the distribution for all deaths and specifically for accidental deaths.

**Table 1:** Child Health Epidemiology Reference Group (CHERG)'s estimates of the number of deaths in China in the year 2010 by province among neonates, post–neonatal infants, children aged 1–4 y and 0–4 y (columns 2–5) and the number of those deaths attributable to accidents (columns 6–9)*

	Total number of deaths in each province by age group				Number of accidental deaths in each province by age group			
**Province**	**Neonatal**	**1 month – 1 year**	**1–4 years**	**Total**	**Neonatal**	**1 month – 1 year**	**1–4 years**	**Total**
Anhui	10 165	4638	2771	17574	407	500	880	1787
Beijing	370	97	118	585	15	16	45	75
Chongqing	3100	1256	880	5235	124	149	293	566
Fujian	3715	1422	1074	6212	149	176	365	690
Gansu	3589	1610	984	6183	144	176	315	634
Guangdong	6636	2097	2025	10757	265	298	731	1295
Guangxi Zhuang AR	10 098	4569	2761	17 428	404	496	880	1780
Guizhou	12 927	7504	3197	23 628	517	650	908	2076
Hainan	1703	791	461	2955	68	84	145	298
Hebei	13 542	6418	3640	23 600	542	670	1138	2349
Heilongjiang	4673	2343	1229	8245	187	233	375	795
Henan	9090	3234	2685	15009	364	423	935	1721
Hubei	5311	2145	1509	8965	212	255	503	970
Hunan	7487	3024	2128	12639	299	360	709	1368
Jiangsu	4109	1246	1266	6621	164	182	463	809
Jiangxi	9304	4463	2489	16256	372	461	774	1607
Jilin	1119	327	348	1794	45	49	129	222
Liaoning	2530	924	742	4195	101	119	256	476
Neimenggu (Inner Mongolia) AR	2703	1223	739	4666	108	133	236	476
Ningxia Hui AR	1475	744	387	2606	59	74	118	250
Qinghai	1278	607	343	2228	51	63	107	222
Shaanxi (Qin)	6049	3165	1564	10778	242	303	468	1012
Shandong	8913	3485	2560	14958	357	425	863	1644
Shanghai	275	61	91	426	11	11	36	58
Shanxi (Jin)	5608	2726	1493	9828	224	278	461	964
Sichuan	24 624	14 329	6083	45 035	985	1238	1726	3950
Tianjin	360	92	115	567	14	15	44	73
Xinjiang Wei AR	7701	4189	1959	13848	308	386	575	1269
Xizang (Tibet) AR	809	430	208	1447	32	41	62	135
Yunnan	8480	4169	2248	14897	339	422	691	1452
Zhejiang	3256	1209	950	5414	130	153	326	609
**Age–specific total for China**	**180 998**	**84 535**	**49 048**	**314 581**	**7240**	**8838**	**15 554**	**31 633**
**% of total for China**	**100.0%**	**100.0%**	**100.0%**	**100.0%**	**4.0%**	**10.5%**	**31.7%**	**10.1%**

*These CHERG estimates [3] are based on models for China for the year 2008 that were explained in detail by Rudan et al. [4].

To achieve *the second aim* – ie, to estimate the relative contribution of accidents to all child deaths in each of these age groups, we simply applied all previously developed models [4] to the number of livebirths in China in 2010 and U5MR in 2010, both of them provided from the same sources as in the previous study [1,3,12,13]. We then divided the total number of estimated accidental deaths by the total number of deaths due to all causes in 3 major age groups: neonates (0–28 days), post–neonatal infants (1–11 months), and children aged 1–4 years. We also performed these calculations for the whole period 0–4 years and presented the results in **Table 1**.

**Figure 1:**
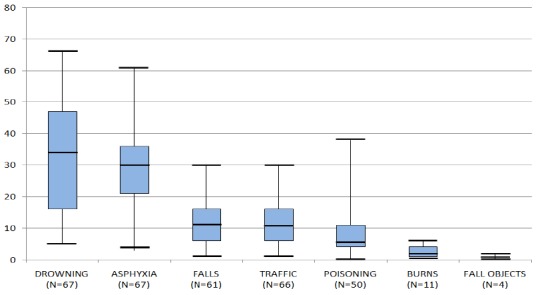
Box and whiskers plot of the proportions (Y–axis) of accidental childhood deaths in China in 2010 by specific causes (X–axis): medians, inter–quartile ranges, maximum and minimum value observed in the studies that provided adequate information. The number of studies available for each cause ranged from 4 to 67 and is presented below X–axis.

To achieve *the third aim* – to define a specific cause to all accidental childhood deaths in China – we required additional information on the specific causes of accidental deaths, which we did not acquire in our previous study on the causes of child deaths in China [4]. To obtain this information, we first reviewed all 206 multi–cause studies published 2000–2008 to identify those that reported accidents as causes of child deaths and we identified 106 such studies. Then, we reviewed those studies for a more specific breakdown of causes of such deaths, but only 5 of 106 studies provided this information. This necessitated a new review of the literature in search of single–cause studies on accidental childhood deaths that provided a detailed breakdown by cause. We repeated the search of the literature using the same procedure as in our previous paper [4], with the only difference that we added the search terms “accidents” and/or “injuries”. This allowed us to identify 71 further studies, nearly all of them from CNKI and Wan Fang databases. Adding the five multi–cause studies from the original review [4], this allowed us to reach a total of 76 studies with useful information. Among them, 67 reported drowning and asphyxia as specific causes, 66 reported traffic accidents, 61 reported falls, 50 reported at least one death due to poisoning, 11 reported burns and 4 reported falling objects. All other causes were sporadic and mentioned only as a rare observation in a single study. From all 76 studies it was possible to compute proportional contributions of these causes to all accidental deaths. For each specific cause we presented median proportion, inter–quartile range (IQR) and maximum and minimum observed percentage in a box–and–whiskers plot in **Figure 1**.

**Figure 2:**
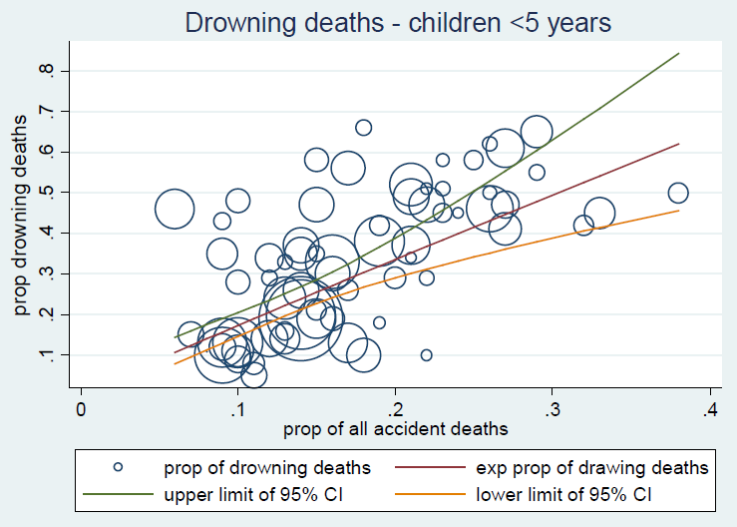
Epidemiological modeling of the association between the most significant predictor variable (proportion of accidental deaths in all child deaths in each study; X–axis) and criterion variable (proportion of deaths caused by drowning in all accidental deaths; Y–axis). Data points represent studies with available information and the size of the “bubbles” is proportional to the total number of child deaths observed in each study. The regression line with upper and lower limit of 95% confidence interval is shown across the range of data.

**Figure 3:**
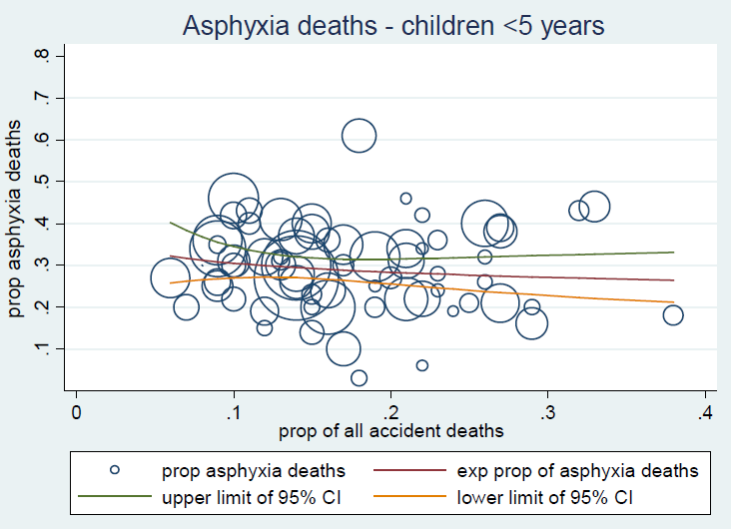
Epidemiological modeling of the association between the most significant predictor variable (proportion of accidental deaths in all child deaths in each study; X–axis) and criterion variable (proportion of deaths caused by asphyxia in all accidental deaths; Y–axis). Data points represent studies with available information and the size of the “bubbles” is proportional to the total number of child deaths observed in each study. The regression line with upper and lower limit of 95% confidence interval is shown across the range of data.

**Figure 4:**
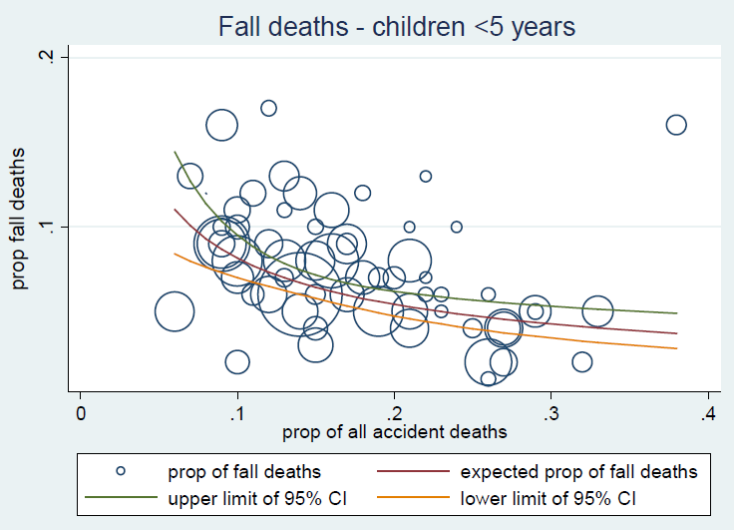
Epidemiological modeling of the association between the most significant predictor variable (proportion of accidental deaths in all child deaths in each study; X–axis) and criterion variable (proportion of deaths caused by falls in all accidental deaths; Y–axis). Data points represent studies with available information and the size of the “bubbles” is proportional to the total number of child deaths observed in each study. The regression line with upper and lower limit of 95% confidence interval is shown across the range of data.

**Figure 5:**
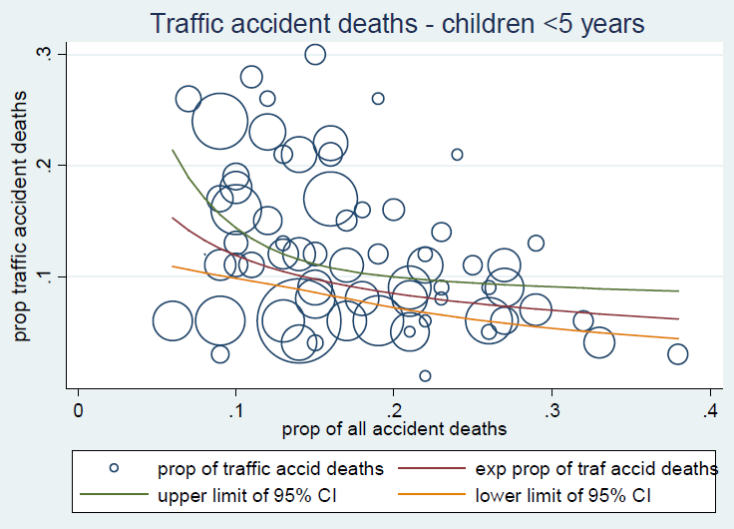
Epidemiological modeling of the association between the most significant predictor variable (proportion of accidental deaths in all child deaths in each study; X–axis) and criterion variable (proportion of deaths caused by traffic accidents in all accidental deaths; Y–axis). Data points represent studies with available information and the size of the “bubbles” is proportional to the total number of child deaths observed in each study. The regression line with upper and lower limit of 95% confidence interval is shown across the range of data.

**Figure 6:**
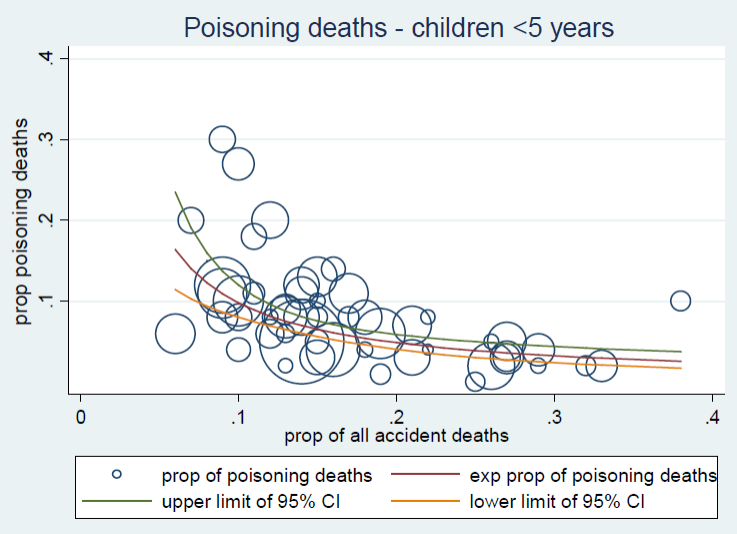
Epidemiological modeling of the association between the most significant predictor variable (proportion of accidental deaths in all child deaths in each study; X–axis) and criterion variable (proportion of deaths caused by poisoning in all accidental deaths; Y–axis). Data points represent studies with available information and the size of the “bubbles” is proportional to the total number of child deaths observed in each study. The regression line with upper and lower limit of 95% confidence interval is shown across the range of data.

Finally, *the fourth aim* of our study was to investigate if there are any significant context–related predictors of the proportional contribution of specific causes to the overall number of deaths due to accidents. This aim should inform us whether we can predict the proportional contribution of different specific causes if we have other information about the context. We could specify three different predictors that were available at the provincial level: (i) overall U5MR; (ii) U5MR that is due to all accidental deaths; and (iii) proportional contribution of accidental deaths to all deaths in each province. We used regression analysis and three separate models to explore whether any of these three predictor variables are significantly associated with proportion of any specific cause in all accidental deaths. We also added the fourth model, where we used multivariate design to account for all three predictors at the same time. We used those four models to predict the proportional contribution of the five specific causes with sufficient information available: drowning, asphyxia, traffic accidents, falls and poisoning. In performing these analyses, we followed all procedures as detailed in our previous paper [4]. We presented a summary of the four models applied to five specific causes of accidental deaths in **Table 2** and **Figures 2** to **6**.

**Table 2:** A summary of results of epidemiological modeling: association between proportion of deaths due to each specific accidental cause (criterion variable) and four different predictor variables

Model	Coefficient (β)	Adjusted R^2^
**Cause of death: drowning (n = 67 studies)**		
ln (U5MR)	0.05	–0.01
ln (U5AMR)	0.25	0.10
ln (%ACC in total)	0.95	0.36
ln (all three predictors)	–0.81	0.35
**Cause of death: asphyxia (n = 67 studies)**		
ln (U5MR)	0.006	–0.015
ln (U5AMR)	–0.02	–0.013
ln (%ACC in total)	–0.11	–0.002
ln (all three predictors)	0.35	–0.03
**Cause of death: falls (n = 61 studies)**		
ln (U5MR)	–0.12	0.01
ln (U5AMR)	–0.24	0.14
ln (%ACC in total)	–0.59	0.23
ln (all three predictors)	1.39	0.24
**Cause of death: traffic accidents (n = 67 studies)**		
ln (U5MR)	–0.43	0.24
ln (U5AMR)	–0.43	0.34
ln (%ACC in total)	–0.49	0.10
ln (all three predictors)	–1.02	0.32
**Cause of death: poisoning (n = 50 studies)**		
ln (U5MR)	–0.05	–0.02
ln (U5AMR)	–0.29	0.10
ln (%ACC in total)	–1.01	0.37
ln (all three predictors)	0.03	0.34

U5MR – under–five mortality rate (all deaths included) reported in each study, U5AMR – under–five mortality rate based on accidental deaths reported in each study, %ACC in total – the proportion of accidental deaths in all deaths in each study, β – regression coefficient, R^2^ – the proportion of variance in the criterion variable explained by each predictor variable

Having completed the analyses towards the third and the fourth aim, it was possible to predict the absolute number of accidental deaths due to each of the specific causes in China in 2010 in two main ways: (i) using median proportional contributions and IQRs from all available studies and applying them to estimates of all accidental deaths in China; or (ii) using the model that explained the largest proportion of variance and applying it to the entire Chinese population. We presented a comparison of these two approaches in **Table 3**.

**Table 3:** Estimates of the number of child deaths in China in 2010 attributable to different causes of accidental deaths*

Causes of accidental death in children aged 0–4 y in China in 2010	Based on median (with IQR)	No. (deaths)	Based on model (with 95% CI)	No. (deaths)
All accidental deaths	100%	31 633	100%	31 633
Drowning (n = 67 studies)	34% (17–47%)	10755 (5378–14867)	18% (16–20%)	5694 (5061–6327)
Asphyxia (n = 67 studies)	30% (21–36%)	9490 (6643–11 388)	30% (26–35%)	9490 (8224–11 072)
Falls (n = 61 studies)	11% (6–16%)	3480 (1898–5061)	8% (7–10%)	2531 (2214–3163)
Traffic accidents (n = 66 studies)	11% (6–16%)	3480 (1898–5061)	12% (10–15%)	3796 (3163–4745)
Poisoning (n = 50 studies)	6% (4–11%)	1898 (1265–3480)	10% (8–12%)	3163 (2531–3796)
Burns (n = 11 studies)	2% (1–4%)	633 (316–1265)	N/A	N/A
Falling objects (n = 4 studies)	1% (0–2%)	316 (0–633)	N/A	N/A

IQR – interquartile range, CI – confidence interval, N/A – not applicable

*The estimates are based on the number of studies that varied between 4 (for falling objects) and 67 (for drowning and asphyxia). The estimates are derived from medians and inter–quartile ranges (columns 2–3) and from epidemiological models with the highest proportion of variance explained and 95% confidence intervals (columns 4–5).

## RESULTS

Our paper presented the time series with the proportional contribution of major causes of child deaths in China from 2000–2008 [4]. Using the same methods and applying the U5MR and the number of livebirths for the year 2010 for China [3,4,12,13] we arrived to the estimates presented in **Table 1**. There were 314 581 deaths in children aged 0–4 years in China in 2010, and we estimated that 31 633 (10.1%) were due to accidents and injuries. There were 7240 deaths in neonates (4.0% of all neonatal deaths), 8838 deaths in post–neonatal infants (10.5% of all post–neonatal infant deaths) and 15 554 deaths in children 1–4 years (31.7% of all deaths in 1–4 years). This analysis addressed the first and second aim of this study.

**Figure 1** shows the box and whiskers plot of the proportions (Y–axis) of accidental childhood deaths in China in 2010 by specific causes (X–axis): medians, inter–quartile ranges (IQR), maximum and minimum value observed in the studies that provided adequate information. The number of studies available for each cause ranged from 4 to 67. Based on medians and IQR, drowning was the leading specific cause with 34% (IQR 17–47%) of all accidental deaths, followed by asphyxia (30%; IQR 21–36%), falls (11%; IQR 6–16%), traffic accidents (11%; IQR 6–16%), poisoning (6%; IQR 4–11%), burns (2%; IQR 1–4%) and falling objects (1%; IQR 0–2%). Applied to the total number of accidental deaths (31 633), this translates into 10755 (5378–14 867) deaths attributable to drowning, 9490 (6643–11 388) to asphyxia, 3480 (1898–5061) to falls, 3480 (1898–5061) to traffic accidents, 1898 (1265–3480) to poisoning, 633 (316–1265) to burns and 316 (0–633) to falling objects. This analysis addressed the third aim of the study.

We then tried to learn more from the information available in the 76 studies. We were interested in whether any context–specific variables may accurately predict the distribution of specific causes. From each study, we extracted three predictors that were universally available: local U5MR due to all child deaths, local U5MR that was attributable to accidental deaths only, and the ratio between the latter and the former, ie, the proportional contribution of accidental deaths to all child deaths. We also developed a model that took into account all three variables. These variables were useful, because they were readily available for all provinces and for China as a whole. **Table 2** presents a summary of results of epidemiological modeling, in which association between proportion of deaths due to each specific accidental cause (criterion variable) and four different predictor variables was explored. It was clear that the third model, based on the ratio between U5MR due to accidents and U5MR due to all deaths, was generally the most predictive. In **Figures 2** to **6**, we present the association between this predictor and the proportion of accidental deaths due to drowning, asphyxia, traffic accidents, falls and poisoning. Data points in these figures represent studies with available information and the size of “bubbles” is proportional to the total number of child deaths observed in each study. The regression line with upper and lower limit of 95% confidence interval is shown across the range of data.

We then applied these models to the entire population of China. This implicated asphyxia as the leading cause of accidental deaths in China in 2010, with 9490 deaths (95% CI 8224–11 072), followed by drowning (5694 deaths; 95% CI 5061–6327), traffic accidents (3796 deaths; 95% CI 3163–4745), poisoning (3163 deaths; 95% CI 2531–3796) and falls (2531 deaths; 95% CI 2214–3163). Together, these 5 causes explain about 80% of all accidental deaths when modeling is primarily used, while the seven causes with available medians explain more than 95% when the analysis is based purely on medians from the 76 available studies.

**Table 3** shows the differences between two sets of estimates when the two approaches are used. When modeling is used instead of medians, the role of asphyxia and traffic accidents is largely unchanged. However, drowning is revised sharply downwards, although remaining the second most important cause. Falls are also revised downwards, while poisoning is revised upwards.

## DISCUSSION

Reduction in global child mortality is a leading political priority and accidental deaths will soon emerge as one of the main challenges. In this paper we provided a detailed breakdown of causes of accidental deaths in a large middle–income country. An important finding of our study is that, wherever the share of accidental deaths among all child deaths is high, drowning is more likely to be the leading cause; asphyxia seems to be equally important in all contexts, while traffic accidents, poisoning and falls are relatively more important in contexts where the overall share of accidents to all child deaths is low. There are differences in estimates based on medians and modeling. This may be explained by a predominance of studies from the areas in which the proportion of accidental deaths in all child deaths is high. It is not surprising that such contexts would attract more studies, and our analyses showed that this would favor drowning over other causes of death. This is why we believe that the estimates based on modeling are more accurate and robust. We recommend that the estimates based on modeling are considered as the more relevant ones for the whole China.

Knowledge on magnitude is the first step in the public health approach towards reducing the number of deaths from childhood accidents and unintentional injuries [14]. Without this baseline data at the national level on the profile of specific causes in children, awareness of the problem will be very limited, hindering the development of national policies [15]. Injury prevention policies are crucial for guiding preventative efforts to reduce deaths from unintentional injuries [16]. Estimating the mortality burden from accidents and injuries for children aged less than five years can highlight areas that need to be improved to generate the relevant information for policy formulation. Funding and attention in the area of unintentional injuries has not been equivalent to that of communicable and non–communicable diseases [9]. It was estimated that in the period between 2006–2007 only 1% of the WHO’s budget and an additional 1% from other sources were provided for the field of injury control and prevention [17]. Perceptions that unintentional injuries cannot be avoided also need to be challenged [18]. Interventions that have been tracked to monitor progress towards MDG4 and MDG 5 have also neglected interventions that focused on reducing preventable deaths from injuries [3,8].

Aggregating mortality data for low and middle–income countries can increase the awareness of the number of deaths caused by unintentional injuries. However, as highlighted in this study, there is a lack of country level data that underpins mortality estimates. Individual countries, and even areas within a country, can differ with regards to the leading mechanisms causing unintentional injuries, as exemplified by the range of mortality rates presented in this review [11]. For countries signatory to the Convention on the Rights of the Child, it is essential that there is greater awareness of the burden caused by unintentional injuries and focus on its redress [19]. Low and middle–income countries need to use a wide range of low cost already established information systems to collate mortality data for unintentional injuries [14,20]. Suggestions have been made that in countries where mortuaries are used to store bodies, this could be a source of injury data [20]. For instance in the year 1996 in Kumasi, Ghana, improvements were made through training of the nurses to help aid with the accurate recording of the mechanism of injury and the body region affected [15]. A review in 2006 of this mortuary showed that reporting was still high even after funding had ceased, and additional unintentional mechanisms had begun to be collected and reported [21]. Previously established demographic surveillance sites could also be used to collect data on mortalities due to injuries. Community based studies are needed to supplement these sources of data as not all deaths will reach the mortuary or be included in a surveillance sites [22,23].

Moreover, improvements need to be made with the reporting of coding that has been used to determine unintentional deaths. Separation of mechanisms that are sometimes placed into the category of ‘other unintentional’ deaths is required to allow within and country comparisons [20]. Some authors suggest that adherence to a strict division between intentional and unintentional accidents and injuries may result in common risk factors being missed [24]. In China, there seems to be a reasonably uniform system of accidents and injuries which is being used to attribute the specific cause of deaths in single–cause studies, but that system does not always easily translate into ICD–10 classification, or in classification used in other countries. There is uncertainty over the deaths caused by asphyxia in China, which seem to cause a large proportion of all deaths and it is possible that they therefore include SIDS – “*sudden infant death syndrome*”. However, it was not possible to learn more about this important issue from the studies themselves and further work would be needed to disentangle and characterize this category. Other categories – such as drowning, traffic accidents, falls, poisoning, burns and falling objects – are much easier to translate to other classifications.

Some middle–income countries, such as Viet Nam, have already moved towards tackling childhood unintentional injuries systematically, though specific policies. The establishment of a National Policy on Injury Prevention has resulted in an increased awareness to the problem of unintentional injuries in children in Viet Nam and has given some direction for preventative efforts [25]. Educational campaigns, swimming lessons, legislation and enforcement of helmet wearing for the population aged over 6 years and creating safer homes, schools and community environments have all been implemented to some degree throughout the country [25]. Further improvements need to be achieved in terms of enforcing current legislations, and extending the helmet legislation to those aged less than 6 years, and information systems for collecting mortality data [25]. We believe that China is very well suited to follow and make further important progress in the reduction of child mortality – which has been the most striking and successful among all the low and middle–income countries over the past two decades.
